# Editorial: Neuroendocrine tumors of the gastrointestinal tract, liver, and pancreas: current management and treatment strategies

**DOI:** 10.3389/fsurg.2023.1207630

**Published:** 2023-05-09

**Authors:** Alexandros Giakoustidis, Alejandro Serrablo, Dimitrios Giakoustidis, Ioannis Moschos, Vasileios N. Papadopoulos, Christos Toumpanakis

**Affiliations:** ^1^ 1st University Surgical Department, Papageorgiou Hospital, School of Medicine, Aristotle University Thessaloniki, Thessaloniki, Greece; ^2^ Department of Surgery, Hospital Universitario Miguel Servet, Zaragoza, Spain; ^3^ International Hellenic University, Thessaloniki, Greece; ^4^ Centre for Gastroenterology, Neuroendocrine Tumour Unit, ENETS Centre of Excellence, Royal Free Hospital, London, United Kingdom

**Keywords:** neuroendocrine tumor, NET, pancreas, gastrointestinal, liver, chromogranin, prognostic markers

**Editorial on the Research Topic** Neuroendocrine tumors of the gastrointestinal tract, liver, and pancreas: current management and treatment strategies

Neuroendocrine neoplasms (NETs) are rare and heterogeneous tumors that are phenotypically similar and originate from the diffuse neuroendocrine cell system. They demonstrate a rising prevalence and incidence which can be partly attributed to the more in-depth understanding of these neoplasms nowadays, but also to the advent and integration of more advanced diagnostic means (1). NETs exhibit slow growth and often an absence of specific symptoms, which is one reason for a belated diagnosis until it is at an advanced stage when overt symptoms could develop. Among sites of origin, gastroenteropancreatic NETs (GEP NETs) represent the commonest subtype, accounting for nearly 60% of all NETs. Among these, small bowel- (SBNEN) and pancreatic-NENs (pNEN) are the most frequent (2–5).

NETs exhibit a variable biologic behavior. They could either be classified as tumors with a “benign” pattern of characteristics without remarkable disease progression and with an excellent prognosis, but there are also tumors that are malignant, associated with an aggressive course, poor prognosis, and a very limited life expectancy.

Therefore the complexity and variability of NETs dictate a wider and better understanding of the current diagnostic and strategic approach and also the determination of the optimal treatment, including an accurate selection of surgical candidates. We are extremely proud and happy for the success of this special issue on NETs as there has been a great response from authors around the world covering, via their accepted publications, aspects of all hot topics. It has been a privilege for me personally to guest edit this special issue with the collaboration of a team of editors including Professor V. Papadopoulos, Professor A. Serrablo, Professor D. Giakoustidis, assistant Professor I. Moschos, and Professor C. Toumpanakis who is a leading figure London-based Gastroenterologist specialist in managing NETs.

The diagnostic cascade should be initiated once there is a clinical suspicion of a NET. Koffas et al. have provided a very thorough review of diagnostic work-up and levels of advancement in the diagnosis of gastroenteropancreatic neuroendocrine neoplasms. The initial work-up involves the assessment of serum Chromogranin A and, in selected patients, the measurement of gut peptide hormones. The description of the measurement of multiple NEN-related transcripts or the detection of circulating tumor cells has enhanced our current diagnostic armamentarium and appears to perhaps even supersede historical serum markers such as Chromogranin A.

Interestingly, Li et al. present two novel nomogram models based on sex, age, and serum NSE levels to preoperatively predict the histologic grades in GEP-NETs to assist in clinical decision-making. Additionally, Li et al. report on risk factors and predictive score models for early recurrence following curative surgery for patients with poorly differentiated gastrointestinal neuroendocrine neoplasms. As they describe in their study, tumor location, preoperative ALP, and LNR are highlighted as independent factors associated with early recurrence, and the risk-scoring model developed based on these three factors appears to exhibit superior predictive efficiency.

Furthermore, Prisciandaro et al. offer a detailed overview of the current landscape of biomarkers in NETs with high-grade features with a specific focus on those harboring potentially therapeutic targets in the advanced setting.

An interesting parameter of Carcinoid heart disease (CHD) which is a consequence of neuroendocrine tumors releasing 5-hydroxytryptamine (5-HT) into the systemic circulation and affecting right heart valves, causing fibrosis and eventually right heart failure is presented by Shah et al. Its surgical correction with valve replacement surgery improved 5-HIAA levels and also improved liver function and hepatic IVC diameter.

Moving into the surgery section, Mou et al. having analyzed data from 536 patients report a potentially improved survival with primary tumor resection in pancreatic neuroendocrine patients with liver metastases. However, when primary tumor resection was combined with synchronous liver metastasis resection, it was not related to a better survival benefit.

On the other hand, Ye et al. focus on patients with non-functional pancreatic NETs smaller or equal to 2 cm as these patients exhibit different biological behaviors which correlate with different prognostic impacts of surgery. The authors' suggestion is that as long as distant metastasis does not occur and the grade is well–moderately differentiated, these patients will not benefit from surgery regardless of lymph node metastasis. However, when local invasion appears in this group of patients, they advise performing surgery. The same advice also goes for patients with a tumor of poorly differentiated or undifferentiated grade or those with distant metastases as surgery could be of benefit.

Interesting and rare cases are always in demand for discussion, and this is the case with the description of a composite Paraganglioma of the Celiac Trunk in a comprehensive literature review by Tzikos et al.. As we are moving with great speed into enhanced recovery after surgery (ERAS) protocols worldwide, Su et al. give us a detailed overview of what is happening in the world in this field with a bibliometric and visualized study on the global states and hotspots of ERAS research in the last two decades.

Lastly, Opalińska et al. highlight the value of peptide receptor radionuclide therapy as a neoadjuvant treatment in the management of primary inoperable neuroendocrine tumors.
